# The impact of induction chemotherapy on the dosimetric parameters of subsequent radiotherapy: an investigation of 30 consecutive patients with locally-advanced non-small cell lung cancer and modern radiation planning techniques

**DOI:** 10.1186/s13014-015-0332-9

**Published:** 2015-01-31

**Authors:** Jonathan D Grant, Angela Sobremonte, Evangeline Hillebrandt, Pamela K Allen, Daniel R Gomez

**Affiliations:** Department of Radiation Oncology, MD Anderson Cancer Center, 1515 Holcombe Blvd Unit 097, Houston, TX 77030 USA

## Abstract

**Purpose:**

To investigate the influence of induction chemotherapy (ICT) on dosimetric outcomes in patients with inoperable non-small cell lung cancer (NSCLC) treated with definitive chemoradiation (CRT).

**Materials and methods:**

30 patients with inoperable stage II-III NSCLC treated with 2–4 cycles of ICT followed by definitive CRT to ≥ 60 Gy were selected. Tumor response to chemotherapy was scored by RECIST criteria. Treatment plans based on tumor extent prior to chemotherapy were generated based on equivalent planning constraints and techniques as the original post-chemotherapy plans. Dosimetric parameters predictive of toxicity for lung, esophagus, heart, and spinal cord were compared amongst the pre- and post-ICT plans.

**Results:**

The majority of patients (70%) experienced an overall reduction in GTV size between the pre-ICT imaging and the time of simulation. Comparing pre-and post-ICT diagnostic imaging, 5 patients met the RECIST criteria for response, 23 were classified as stable, and 2 experienced disease progression on diagnostic imaging. Despite a significantly reduced GTV size in the post-ICT group, no systematic improvements in normal tissue doses were seen amongst the entire cohort. This result persisted amongst the subgroup of patients with larger pre-ICT GTV tumor volumes (>100 cc^3^). Among patients with RECIST-defined response, a significant reduction in lung mean dose (1.9 Gy absolute, median 18.2 Gy to 16.4 Gy, p = 0.04) and V_20,_ the percentage of lung receiving 20 Gy (3.1% absolute, median 29.3% to 26.3%, p = 0.04) was observed. In the non-responding group of patients, an increased esophageal V_50_ was found post-chemotherapy (median 28.9% vs 30.1%, p = 0.02).

**Conclusions:**

For patients classified as having a response by RECIST to ICT, modest improvements in V_20_ and mean lung dose were found. However, these benefits were not realized for the cohort as a whole or for patients with larger tumors upfront. Given the variability of tumor response to ICT, the *a priori* impact of induction chemotherapy to reduce RT dose to normal tissue in these patients is minimal in the setting of modern treatment planning.

## Introduction

In the treatment of non-small cell lung cancer (NSCLC), induction chemotherapy (ICT) is commonly used as a means of cytoreduction to reduce subsequent radiotherapy field size or to convert a previously unresectable lung tumor to resectability. The actual impact, however, of this approach in improving dosimetric parameters to normal tissues using modern techniques in clinical practice is not well studied. ICT has not been shown to confer survival advantage in a sequential approach with concurrent chemoradiotherapy (CRT) and confers added toxicity and treatment time [1,2].

Given these costs, we sought to investigate dosimetric impact of ICT in 30 sequential patients treated with a homogeneous and well-accepted regimen of ICT followed by definitive CRT using modern planning techniques. The effects of ICT on target and normal tissue dosimetric variables were quantified in this population by comparing treatment plans generated from pre- and post-chemotherapy volumes, both as an entire cohort and stratified by RECIST response.

## Methods and materials

### Patient characteristics

Appropriate MD Anderson Institutional Review Board approvals were obtained. We retrospectively reviewed our institutional records from 2007 to 2010 and selected 30 consecutive patients that met the following criteria: (a) Stage II-III NSCLC (according to the 7th edition of the American Joint Committee on Cancer staging manual [3]), (b) treated with an ICT regimen 2–4 cycles of a platinum/taxol doublet, (c) for whom a diagnostic CT scan prior to initiation of chemotherapy was available for review, (d) who were then treated with definitive CRT to a radiation dose ≥ 60 Gy. A summary of patient and disease characteristics is presented in Table 1.

**Table 1:** **Baseline patient and treatment characteristics**

**Characteristic**	**Patients (n = 30)**
Sex	
Female	12
Male	18
Ethnicity	
Caucasian	26
Other	4
Tumor histology	
Nonsquamous	14
Squamous	16
T Stage	
T1	3
T2	12
T3	5
T4	10
TX	
N Stage	
N0	2
N1	3
N2	17
N3	8
Disease stage	
IIB	2
IIIA	11
IIIB	17
Response to ICT	
Partial	3
Progressive	2
Stable	27
ICT end to RT start (days)	
Median	18
Range	1-80
RT dose (Gy)	
Median	70
Range	60-74

*Abbreviations:* *ICT* induction chemotherapy, *RT* radiotherapy.

### Chemotherapy

Patients received ICT for a variety of reasons, including: 1) cytoreduction in an attempt to render the patient’s disease operable or reduce radiation toxicity (18 patients) and 2) questionable metastatic disease on imaging, such that chemotherapy was given to allow patients to “declare” themselves as having true locoregional versus metastatic malignancy prior to a definitive course of radiation therapy (5 patients). The reason for induction chemotherapy administration was not explicitly stated in 7 patients. All patients received 2–4 cycles of a platinum/taxol doublet. 15 patients received carboplatin-based therapy, and 15 received cisplatin-based therapy. 23 patients were treated with taxotere, and 7 with paclitaxel. 15 patients received 2 cycles of ICT, 11 were treated with 3 cycles, and 4 received 4 cycles. The median time interval from the final day of chemotherapy administration to RT start was 1.1 months (range 0.1-3.5 months).

The pre- and post-ICT diagnostic CT scans were reviewed and scored by a single investigator (JG) according to the Response Evaluation Criteria for Solid Tumors (RECIST) guidelines [4]. Tumor size was obtained by direct measurements on the CT scans. In accordance with the RECIST grading system, complete response was defined as disappearance of all target lesions; a partial response was represented by a 30% or greater decrease in the sum of the longest diameter of target lesions; and progressive disease was an increase of 20% or more in these parameters, or any new sites of disease. Changes not meeting any of these criteria were defined as stable disease. For the purposes of analysis, patients with stable and progressive disease were grouped as non-responders.

### Radiotherapy

Definitive CRT was delivered with intensity modulated radiation therapy (IMRT) in 28 patients, and 3-D conformal therapy in 2 patients. Simulation was performed with 4DCT, and the gross tumor volume including the primary lung tumor and involved lymph nodes was contoured across all phases of the respiratory cycle to encompass motion (internal GTV, or iGTV). Contouring for the delivered post-ICT plans was completed by a variety of attending physicians at our institution. Though individual approaches were varied, standard institutional contouring techniques include nodal levels involved pre-chemotherapy and post-chemotherapy parenchymal volumes. Lung windows were used to define the extent of parenchymal disease, with soft tissue windows to identify mediastinal and lymph node disease. A 6- to 8-mm expansion was applied from the iGTV structure to create the clinical target volume (iCTV, approximating the ITV), which was modified based on surrounding anatomic structures. A 5-mm expansion was added to the CTV to create a planning treatment volume (PTV).

A range of radiation doses were used, based on the preference of the treating physician and the enrollment of patients on dose-escalation protocols. 1 patient was treated to a radiation dose of 60 Gy, 5 received 63 Gy, 6 received 66 Gy, 6 received 70 Gy, and 12 received 74 Gy. Dose per fraction ranged from 1.8 to 2.4 Gy.

### Delineation of pre-ICT tumor volumes

In delimitating the pre-ICT tumor volumes, the diagnostic CT obtained immediately prior to ICT administration were spatially overlaid onto the post-ICT simulation CT dataset. Using anatomic landmarks including bones and cardiopulmonary structures to visually guide contour delination, two investigators (JG and DG) recreated the pre-ICT tumor volume on the post-ICT image set. iCTV and PTV expansions were applied to match the technique used in the post-ICT planning process. Because target volumes were transferred onto the post-ICT dataset, identical normal tissue ROI contours could be preserved despite the difference in setup positioning between the diagnostic and simulation CT scan. Normal lung volumes, which are defined at our institution by subtracting the GTV from the total lung volume, were recalculated for the pre-ICT setting given the difference in GTV size between the datasets.

**Figure 1:**
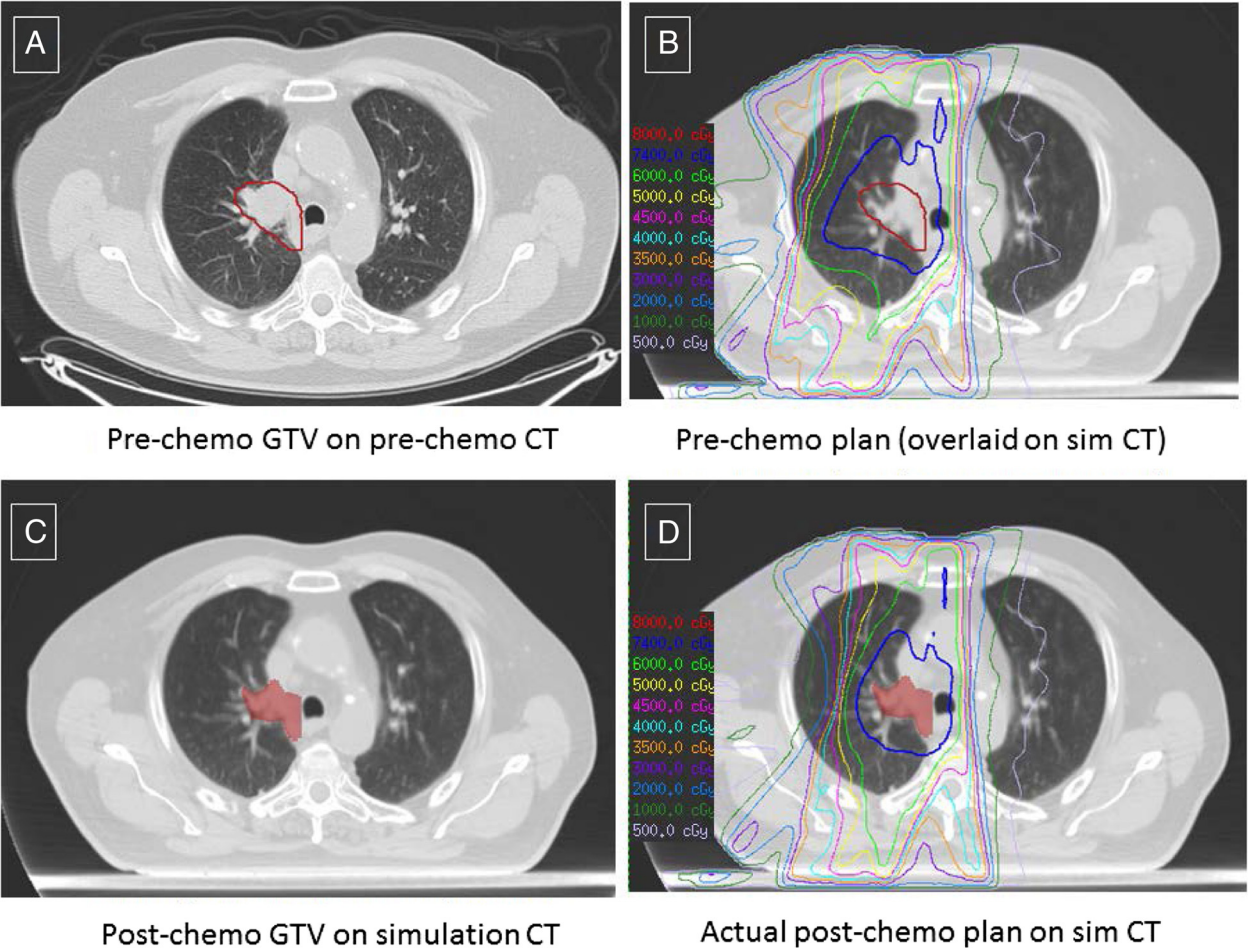
**Gross tumor volumes (GTVs) on CT scans obtained before and after induction chemotherapy in a representative case.** The pretreatment GTV was first contoured on a diagnostic CT scan obtained before chemotherapy **(A)** and overlaid on the simulation CT **(B)**, and a radiation therapy plan was generated based on normal tissue contours on the simulation scan. After chemotherapy, the GTV was outlined on the simulation CT by the treating physician **(C)** and the subsequent radiation treatment was planned according to institutional standards as described in the text **(D)**.

Two medical dosimetrists (AS and EH) then generated radiation plans based on these pre-ICT target volumes, to the same prescription dose and using the same target goals and normal tissue constraints (see Figure 1). For treatment planning, class solutions were applied with templated beam arrangements and planning objectives based on the tumor location and extent. Standard institutional normal tissue dose constraints for concurrent CRT are found in Table 2. Dose was minimized to the surrounding normal tissue ROIs while keeping 95% of the PTV covered by 95% of the prescription dose (PTV_95_ ≥ 95%). In circumstances where a large tumor size precluded the ability to achieving these constraints, target coverage was sacrificed in order to maintain normal tissue constraints, allowing PTV_95_ to serve as single variable for comparison in these cases.

**Table 2:** **Institutional planning constraints for concurrent chemoradiation to the thorax**

**Structure**	**Dose constraint**
Lung	
V_5_	<65%
V_20_	<35%
Mean	≤20 Gy
Heart	
V_50_	<50%
Mean	26 Gy
Esophagus	
V_55_	<50%
Mean	≤34 Gy
Spinal cord	
Maximum	45 Gy
PTV	
PTV_95_ goal	≥95%

In patients where updated institutional planning techniques resulted in significant differences in beam arrangement and overall plan quality, post-chemotherapy plans were also re-planned using current methods consisting of template beam arrangements based on the position of the tumor within the thorax. Dose-volume histogram (DVH) data points for targets and normal structures for both pre-ICT and post-ICT plans were extracted using relational database software [5]. The following parameters were collected: lung V_5_ and V_20_, mean lung dose, maximum spinal cord dose, heart mean, heart V_40_, esophagus mean, esophagus V_50_, and maximum esophageal dose.

### Statistical methods

A Wilcoxon matched-pairs signed-ranks test method was used to determine significant differences between the pre- and post-chemotherapy plans with regards to the GTV, CTV and PTV sizes, dose to 95% of the GTV (GTV_95_), CTV_95_, PTV_95_, and the normal regions of interest as described above. These values were compared across the entire cohort as well as segregated by RECIST classification and pre-chemotherapy GTV size > or ≤ 100 cc^3^. *P* values ≤0.05 were considered to indicate statistically significant differences. Stata/MP version 13.1 (College Station, TX) was used for all statistical tests.

## Results

**Figure 2:**
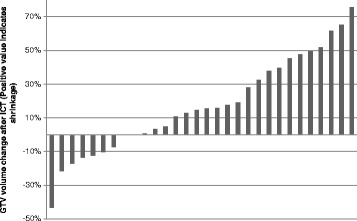
**Differences in contoured GTV size following ICT, displayed as a percentage of pre-ICT volume.** Positive values indicate a GTV that has reduced in size following ICT, and negative values indicate a larger GTV.

Median time from completion of the last chemotherapy course to radiation simulation was 18 days. Comparing pre- and post-ICT diagnostic CT scans by RECIST criteria, 5 (17%) had a partial response, 23 (77%) had stable disease, and 2 (7%) had progressive disease. By absolute GTV volume change between the pre-ICT diagnostic scan and the RT simulation scan, 23 (77%) had unchanged or smaller volumes after chemotherapy (Figure 2), while in 7 (23%) the volume had increased. All normal tissue dose constraints were met in each patient. In 5 patients, a PTV_95_ of ≥95% was not achievable in pre-ICT plans and coverage was thus sacrificed.

**Figure 3:**
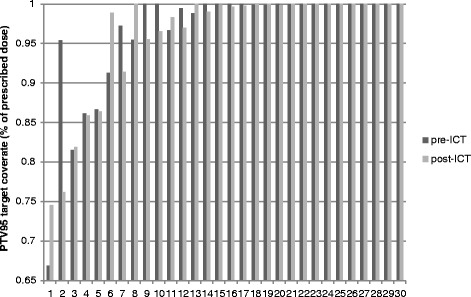
**PTV95 coverage (displayed as a percentage of prescribed dose) for pre-ICT versus post-ICT plans.** 5 patients had initial pre-ICT plans that did not meet target coverage goals, of whom 2 derived benefit from chemotherapy (patients 1 and 6). 2 plans (patients 2 and 7) showed >5% compromised coverage for tumor volumes present following ICT.

**Figure 4:**
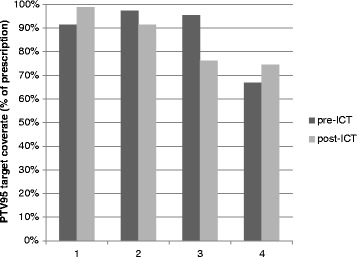
**Four patients showed a >5% difference between pre- and post-ICT PTV95 coverage.** One patient met target coverage goals following ICT, two patients lost acceptable coverage following ICT, and one patient derived benefit but remained below the goal of 95%.

As shown in Figure 3, two patients experienced significantly improved PTV coverage following ICT (approximately 7.5% absolute improvement), one of whom achieved a PTV_95_ ≥ 95%. 2 patients with adequate PTV_95_ coverage on the pre-ICT plan showed significantly decreased target volume coverage on the post-ICT plans. A total of 4 patients with compromised PTV_95_ had a >5% difference between pre-ICT and post-ICT plans, 2 of whom had improved coverage following ICT, and in 2 of whom coverage decreased (Figure 4).

Results of the collective DVH analysis of target volumes, target coverage, and dose to normal tissues are represented in Table 3. No target coverage parameters or normal tissue doses were significantly different before as compared to after ICT. Among the 5 who experienced a RECIST response, a significantly decreased mean lung dose (median 18.2 Gy vs 16.4 Gy, p = 0.04) and lung V_20_ (median 29.3% vs 26.3%, p = 0.04) was seen in the post-chemotherapy plans. In the non-responding group of patients, an increased esophageal V_50_ was found post-chemotherapy (median 28.9% vs 30.1%, p = 0.02). No differences in normal tissue doses were observed when stratifying based on initial pre-ICT GTV volume > or ≤100 cc^3^.

**Table 3:** **Median values for dosimetric variables derived from scans obtained before and after induction chemotherapy**

	**All patients (n = 30)**			**RECIST responders (n = 5)**			**RECIST stable and progressive (n = 25)**			**Pre-ICT GTV >100 cc** ^**3**^ **(n = 21)**			**Pre-ICT GTV ≤100 cc** ^**3**^ **(n = 9)**		
**Variable**	**Pre-ICT**	**Post-ICT**	P*	**Pre-ICT**	**Post-ICT**	P*	**Pre-ICT**	**Post-ICT**	P*	**Pre-ICT**	**Post-ICT**	P*	**Pre-ICT**	**Post-ICT**	P*
GTV size, cm^3^	117	111	**0.02**	117	62	**0.04**	115	115	0.14	134	126	**0.04**	57	40	0.26
CTV size, cm^3^	344	297	**0.03**	350	218	**0.04**	338	338	0.22	372	379	**0.03**	193	160	0.86
PTV size, cm^3^	546	559	0.37	617	376	**0.04**	528	574	0.76	606	621	0.22	312	342	0.51
GTV_95_, %	100	100	0.73	100	100	0.32	100	100	0.99	100	100	0.74	100	100	0.32
CTV_95_, %	100	100	0.15	100	100	0.40	100	100	0.23	100	100	0.36	100	100	0.40
PTV_95_, %	96.7	96.1	0.31	98.6	99.1	0.89	96.5	94.8	0.26	96.5	95.0	0.43	97.0	97.3	0.37
Lung V_5_, %	50.5	51.8	0.31	50.0	51.2	0.14	50.5	52.6	0.68	52.5	52.2	0.20	50.0	51.2	0.95
Lung V_20_, %	29.1	28.9	0.24	29.3	26.3	**0.04**	28.9	29.6	0.86	29.5	29,6	0.61	27.9	27.9	0.11
Lung mean dose, Gy	18.1	18.5	0.45	18.2	16.4	**0.04**	18.1	18.6	0.68	18.2	18.5	0.96	16.1	18.6	0.11
Esophagus mean dose, Gy	28.2	28.4	0.24	26.7	24.0	0.89	28.7	28.4	0.20	31.4	32.3	0.42	22.2	22.2	0.44
Esophagus max, Gy	74.3	76.5	0.56	68.9	67.3	0.22	75.2	76.6	0.27	75.2	76.4	0.97	73.3	76.6	0.21
Esophagus V_50_, %	29.0	28.9	0.08	32.4	20.9	0.22	28.9	30.1	**0.02**	32.1	31.7	0.10	23.8	23.5	0.34
Heart mean dose, Gy	11.1	11.3	0.29	6.3	6.0	0.22	12.6	13.3	0.49	12.6	15.4	0.43	6.6	10.2	0.44
Heart V_40_, %	8.6	7.0	0.63	4.6	3.5	0.17	9.4	8.84	0.86	11.2	10.8	0.70	3.5	6.8	0.50
Spinal cord max, Gy	42.3	42.7	0.19	42.7	41.7	0.5	42.3	42.8	0.25	42.8	43.1	0.33	39.2	41.7	0.37

*Abbreviations*: *GTV* gross tumor volume, *CTV* clinical target volume, *PTV* planning target volume.

*P values calculated with Wilcoxon matched-pairs signed-ranks test. Bolded p values are <0.05 and are taken as statistically significant.

## Discussion

We present herein an investigation into the practical impact of ICT on subsequent radiation planning using modern techniques using IMRT with optimized class-solutions based dosimetry. Target volumes using pre-ICT extent of disease were recreated on the simulation CT scan and compared with the actual post-ICT radiation plans to determine differences. This was performed on 30 consecutive patients for whom complete data and imaging was available. A significant reduction in GTV and CTV volumes was observed across the cohort following ICT; reduction in PTV volumes trended towards but did not reach significance. As an entire cohort these changes did not translate into clinically-relevant differences in normal tissue doses. Amongst patients with a response by RECIST criteria, reductions were seen in lung dose, with increased esophageal dose in RECIST-defined stable and progressive patients. Amongst patients with a larger initial tumor size (<100 cc^3^), greater tumor shrinkage was likewise observed, but this did not translate into any systematically reduced dose to normal tissues.

This is the first study to our knowledge which investigates the impact of ICT on dosimetric outcomes using modern planning techniques including IMRT. A previous report by Jenkins *et al.* evaluated the impact of chemotherapy on subsequent 3D conformal hyperfractionated RT [6]. 38 patients with unresectable stage III NSCLC were treated with an induction chemotherapy regimen consisting of mitomycin C, vinblastine, and carboplatin. A three-field RT technique was used to deliver continuous hyperfractionated accelerated RT to a dose of 56 Gy in 36 fractions given three times a day. 95% of patients experienced quantitative shrinkage following chemotherapy, compared to 70% in our study. Mean reductions after chemotherapy was 37% for GTV and 26% for PTV. The only dosimetric measure which was statistically improved was lung V_20,_ with a mean decrease in 3%. No differences were seen in maximum esophageal or spinal cord dose following chemotherapy.

In contrast, we found no improvement across any dosimetric parameter when evaluating the entire cohort. Among RECIST responders, our findings paralleled those reported by Jenkins *et al.,* with a 3% reduction in V_20._ We also report a median 2 Gy reduction in mean lung dose for responders. Jenkins *et al.* quantified the location of tumor shrinkage, finding the greatest effect along tumor edges opposite the mediastinum, and supporting the negligible sparing of centrally-situated structures near the mediastinum. Our results support this finding, both as an entire cohort and when evaluating only those with a RECIST response; central structures including the esophagus, spinal cord, and heart did not benefit from any dose reduction following chemotherapy. The reduction in lung-specific parameters among RECIST responders supports the patterns of shrinkage along lung parenchymal edges.

In conjunction with prior studies in both NSCLC [6] and SCLC [7], we found the reductions in PTV size to be smaller in magnitude than the corresponding GTV shrinkage. This may be due, in part, to the abutment of tumor against anatomic boundaries from which CTV coverage expansion is reduced. As tumor reduction occurs away from these structures, the CTV expansion that is allowed would effectively enlarge, negating the sparing effects of this shrinkage. This is especially true for mediastinal nodal locations, where vessels, esophagus, and bronchus are typically excluded from CTV expansions.

Differences between the present study and that reported by Jenkins *et al.* include patient selection and planning technique, the former of which highlights some of the limitations of this study. Because tumor size, location, and response to ICT are all unique to individual cases, generalized results are highly dependent on patient selection. Differences in the overall response rate of our cohort compared with that reported by Jenkins *et al.* may thus be due to differences in patient selection and tumor characteristics. In an attempt to minimize bias, we selected the 30 most recent patients for whom all needed datasets were available. The clinical indications for ICT were varied (see Methods and materials), and many actually had PTV_95_ coverage ≥ 95% when plans were generated on the disease extend prior to chemotherapy. Even among those with compromised pre-ICT coverage, however, showed limited benefit (Figure 3). Differences are also likely influenced by our application of modern techniques for RT planning, included IMRT and class-solution algorithms for optimized planning. Compared with the standard 3-field technique employed by Jenkins *et al.,* the plans generated in the present study are likely to lessen the detriment of larger tumor size on surrounding normal structures by improved dosimetric techniques.

Additional limitations of the present study include variation in chemotherapy agents used, timing of treatment, and physician definition of post-ICT target volumes. These factors also render the present study a real-world reflection of clinical practice. The slight variations in physician technique (i.e. 6 versus 8 mm iCTV expansion) were reproduced in the pre-ICT planning process. Though simulation CT scans were 4D and diagnostic CT scans were typically in the breath-hold position, anatomic landmarks were used to accurately depict pre-ICT tumor volume on the simulation CT. Limitations in reconstructing pre-chemotherapy volumes and tumor measurement are previously-described [8,9]. PET-CT based measures of response could not be evaluated as not all patients had this study performed.

As a result of these limited dosimetric improvements and the difficulty in distinguishing peri-tumoral inflammation from microscopic tumor following chemotherapy, Jenkins *et al.* recommend targeting the tumor volumes present prior to ICT in most patients. Given these reported uncertainties, the known lack of survival benefit with the addition of ICT to CRT, and the lack of dosimetric benefit we found amongst the entire cohort, these findings discourage against the general upfront use of ICT for patients with NSCLC. If a significant response by RECIST could be expected, a modest reduction in V_20_ and mean lung dose on the order of 3% and 2 Gy, respectively, could be anticipated. Based on the nomogram reported by Bradley *et al.*, however, these values are not likely to decrease the risk of radiation pneumonitis by more than 5% [10]. Further research to identify patients whose tumors are more biologically likely to respond to ICT would allow this practice to be applied selectively to those who would derive the most benefit.

## Conclusions

In this dosimetric planning study, we found no significant decreases in normal tissue radiation doses following ICT for the cohort as a whole. Although a modest decrease in lung dose was observed in the subset of responding patients by RECIST criteria, treatment response to chemotherapy and the subsequent benefit thereof cannot currently be predicted *a priori*. Given the lack of correlation of ICT response with a substantial clinical or dosimetric benefit in our analysis, we recommend that the decision to institute this treatment in an individual patient prior to definitive chemoradiation also be weighed against the lack of an apparent survival advantage in randomized studies and its associated toxicity.

### Consent

All patients provided consent for the publication of de-identified specimens, images, and other data as part of the overall consent process.
